# Impact of Air Pollution on Residents' Medical Expenses: A Study Based on the Survey Data of 122 Cities in China

**DOI:** 10.3389/fpubh.2021.743087

**Published:** 2021-12-20

**Authors:** Huan Liu, Tiantian Hu, Meng Wang

**Affiliations:** ^1^School of Public Administration, Zhejiang University of Finance and Economics, Hangzhou, China; ^2^School of Political Science and Public Administration, Wuhan University, Wuhan, China

**Keywords:** air pollution, medical expenses, mineral endowment, residents' health, CHARLS

## Abstract

**Background:** With the development of the social economy, air pollution has resulted in increased social costs. Medical costs and health issues due to air pollution are important aspects of environmental governance in various countries.

**Methods:** This study uses daily air pollution monitoring data from 122 cities in China to empirically investigate the impact of air pollution on residents' medical expenses using the Heckman two-stage and instrumental variable methods, matching data from the 2018 China Health and Retirement Longitudinal Study (CHARLS) survey.

**Results:** The study found that poor air quality, measured by the air quality index (AQI), significantly increased the probability of chronic lung disease, heart disease, and self-rated poor health. Additionally, the AQI (with an effect of 4.51%) significantly impacted health-seeking behavior and medical expenses. The medical expenditure effects of mild, moderate, severe, and serious pollution days were 3.27, 7.21, 8.62, and 42.66%, respectively.

**Conclusion:** In the long run, residents' health in areas with a higher air pollution index, indicating poor air quality, is negatively impacted. The more extreme the pollution, the higher the probability of residents' medical treatment and the subsequent increase in medical expenses. Group and regional heterogeneity also play a role in the impact of air pollution on medical expenses. Compared with the existing literature, this study is based on individuals aged 15 years and above and produces reliable research conclusions.

## Introduction

Air pollution is a common problem in human, social, and economic development. Due to its strong mobility, the impact of air pollution on different groups of people is homogeneous and non-discriminatory. In 2018, the World Health Organization (WHO) released the article titled “Ambient (outdoor) air pollution” which discusses four main air pollutants affecting health: particulate matter (PM10, PM2.5), ozone, nitrogen dioxide, and sulfur dioxide. The WHO has issued air quality guidelines to prevent the negative impact of air pollutants on health (1). According to the Bulletin on China's Ecological Environment, 2019 (2), 53.4% of cities at the prefecture level and above failed to meet air quality standards. According to the 2017 Global Disease Burden Report (3) released by the WHO, 161.1 of every 100,000 deaths in China resulted from air pollution. Severe air pollution affects sustainable socio-economic development and poses a major threat to public health. Compared with other environmental pollution factors, residents are more likely to be exposed to air pollution. Serious air pollution increases the prevalence of lung cancer or respiratory diseases (4–6), thus reducing the average life expectancy (7, 8). Theoretically, ecological destruction and environmental pollution reduce quality of life, decrease production efficiency, and affect individual health, increasing the prevalence of related diseases and medical costs (9). Therefore, it is of great practical and theoretical significance to explore air pollution-related health costs from the perspective of individual health and medical expenses.

Compared to indoor air pollution, the causes of outdoor air pollution are complex, such as primary pollutants discharged from industry and secondary pollutants resulting from chemical reactions. Outdoor air pollutants affect a wide range of groups and involve more people. Therefore, this study also attempts to consider outdoor air pollution as the core to investigate its impact on residents' health and medical expenses.

In addition, previous studies (mainly based on short time-dependent observations) have mostly focused on the impact of air pollutants on health and short-term medical expenses (10–12). This study investigated the long-term medical expenses related to poor air quality, including the decline in body function and immunity, and the increase in disease incidence.

## Literature Review

The impact of air pollution on residents' lives has attracted increasing attention and research. The literature on air pollution is rich in aspects such as residents' well-being and environmental migration (13–15). However, there are relatively few studies on the effects of air pollution from the perspective of medical expenses. Theoretically, air pollution has a strong health depreciation effect, indirectly augmenting medical expenses. Therefore, we compare the existing literature based on this theoretical basis.

First, we analyzed the health depreciation effects of air pollution. Studies have shown that with a significant increase in the concentration of air pollutants, such as CO, O_3_, PM2.5, and PM10, the probability of individuals suffering from respiratory diseases, heart disease, or lung cancer also increases significantly (16–18). Cardiovascular disease and mortality risk also increase significantly (19–21). Research on pollutant concentration shows that air pollution significantly increases infant, adult, and elderly mortality and reduces life expectancy (22–27). In addition, air pollution significantly affects mental health. Further, air pollution significantly increases the possibility of residents suffering from depression, anxiety, or cognitive diseases, leading to more serious psychological problems (28–30).

Second, we analyzed the effect of air pollution on medical expenses. Air pollution has caused serious social costs; the reduction of air pollutant concentration can reduce the mortality rate of older persons and increase life expectancy (25, 31), resulting in significant social benefits and economic value (27). Research shows that short- and medium-term air pollution can significantly increase medical expenses. When air pollution is optimized, the total medical expenses can be effectively reduced (32–34). However, the medical costs associated with asthma and other respiratory diseases increase when the concentration of air pollutants increases, especially inhalable particulate matter (35, 36). Additionally, existing studies analyse the medical cost effect of air pollution from the perspective of medical-related expenses, such as drug procurement and air purifiers (32, 37, 38).

Overall, existing studies have generally discussed the effects of air pollution on health and social costs. However, few studies have focused on the effects of air pollution on individual health and medical expenses based on the matching method of micro individual survey data and macro air pollution data. The topic of the individual health costs of air pollution has been neglected. Additionally, most existing studies have evaluated the health cost effect of average air pollution but ignored the important impact of extreme pollution days.

Therefore, based on the existing research, we selected the CHARLS 2018 survey data and 122 regional air pollution (AQI, air quality index)-related statistical data to match and obtain the basic data. Considering the influence of selective bias on medical expenses, we used Heckman's two-stage method to evaluate the probability of medical treatment in one stage and to estimate the medical costs in the other; for example, a medical cost of 0 suggests that the individual has not used medical services. However, some cases in the sample survey had zero medical costs, resulting in estimation bias. Additionally, we used the two-stage instrumental variable method to address the health cost endogeneity of air pollution.

The main contributions and innovations of this study are as follows. First, this study considers respiratory diseases with a higher correlation to air pollution as an important health indicator to investigate the health effects of air pollution and to improve the reliability of related research conclusions. Second, based on the annual AQI statistical indicators, we investigated the impact of extreme pollution on residents' medical expenses to enrich the relevant research on the social cost of air pollution under different intensities. Third, the Heckman two-stage model was used to reduce the estimation bias of the medical consumption effect caused by selective bias. Fourth, considering the regional mineral resource endowment as the instrumental variable, we used the instrumental variable method to solve the endogenous problem of air pollution and to improve the reliability of the research conclusions.

## Methods and Data

### Benchmark Model Construction

First, we established a health impact model for air pollution. We selected health indicators that reflected the incidence of diseases closely related to air pollution. These mainly include chronic lung disease, cancer, heart disease, and self-rated health status, defined as, *y*_*ci*_, and as the core explanatory variables. The regional average AQI in the survey year was selected as the core explanatory variable and recorded as *AQI*_*ci*_. Based on these core variables, we followed the research of Bert and Stephen (39) and Kumar et al. (40) to reduce the influence of other factors. We also controlled for other related factors in the model, including age, gender, education, health, and other individual variables, recorded as *ind*_*ci*_. We concurrently controlled for regional effects, including regional socio-economic development, annual average rainfall, and other characteristic variables, recorded as *city*_*i*_. Based on these variables, we established a health effects model for air pollution as follows:


(1) $$\begin{array}{l} y_{ci}=\alpha_{0}+\alpha_{1}AQI_{ci}+\alpha_{2}ind_{ci}+city_{i}+\varepsilon_{ci} \end{array}$$

We used cross-sectional data from 2018; therefore, we fixed the regional effect *city*_*i*_ in the model to avoid the randomness of the results. ε_*ci*_ represents the random error term, which follows a normal distribution. Thus, we established a medical expenses model for air pollution. When analyzing the impact of air pollution on medical expenses, we usually encounter problems of sample selection bias and endogeneity of air pollution. The Heckman two-stage method is widely used in academia to solve the problem of sample selection bias in medical expenses. Heckman (41) estimated the probability of medical choice in the first-stage selection equation and added the Mills ratio obtained by the regression of the first stage as the explanatory variable in the second-stage outcome equation, to estimate the medical expenses of related samples. Schwiebert (42) developed a Heckman selection model with endogenous covariates. Their model provides an easy-to-interpret measure of the composition of the fully observed sample with respect to unobservable variables. Osiolo and Kimuyu (43) also studied the intervention measures of indoor air pollution using the Heckman sample selection model. Therefore, we used the Heckman two-stage model to reduce bias in the results caused by sample selection in medical expenses. We defined the sample whose medical expenses are >0 as 1, meaning the population in the sample that chose to seek medical attention. However, a sample whose medical expenses are equal to 0 is defined as the behavior of not seeking medical attention. This eliminates the estimation bias caused by the sample that chooses to seek medical attention, but with a medical cost of 0. In the first stage, the limited dependent variable model is as follows:


(2) $$\begin{array}{l} Choice_{ci}=\beta_{1}x_{ci}+\beta_{2}ind_{ci}+city_{i}+\delta_{ci} \end{array}$$

The control variables in model (2) are consistent with model (1), and with the city effect being a fixed effect. Additionally, the core explanatory variable *x*_*ci*_ in model (2) is represented by the annual average *AQI*_*ci*_. Extreme pollution days were also selected as proxies. We classified these days into four categories according to the AQI index value: mild (AQI≥100), moderate (AQI≥150), severe (AQI≥200), and serious pollution days (AQI≥300). Refer to Variable Definition and Data for specific variable definitions. Therefore, when *Choice*_*ci*_= 1, we set the second-stage medical expenses effect model as:


(3) $$\begin{array}{l} Medical\text{\_}\exp ense_{ci}=\chi_{1}+\chi_{2}x_{ci}+\chi_{3}ind_{ci}+city_{i}+\phi_{ci} \end{array}$$

**Figure 1:**
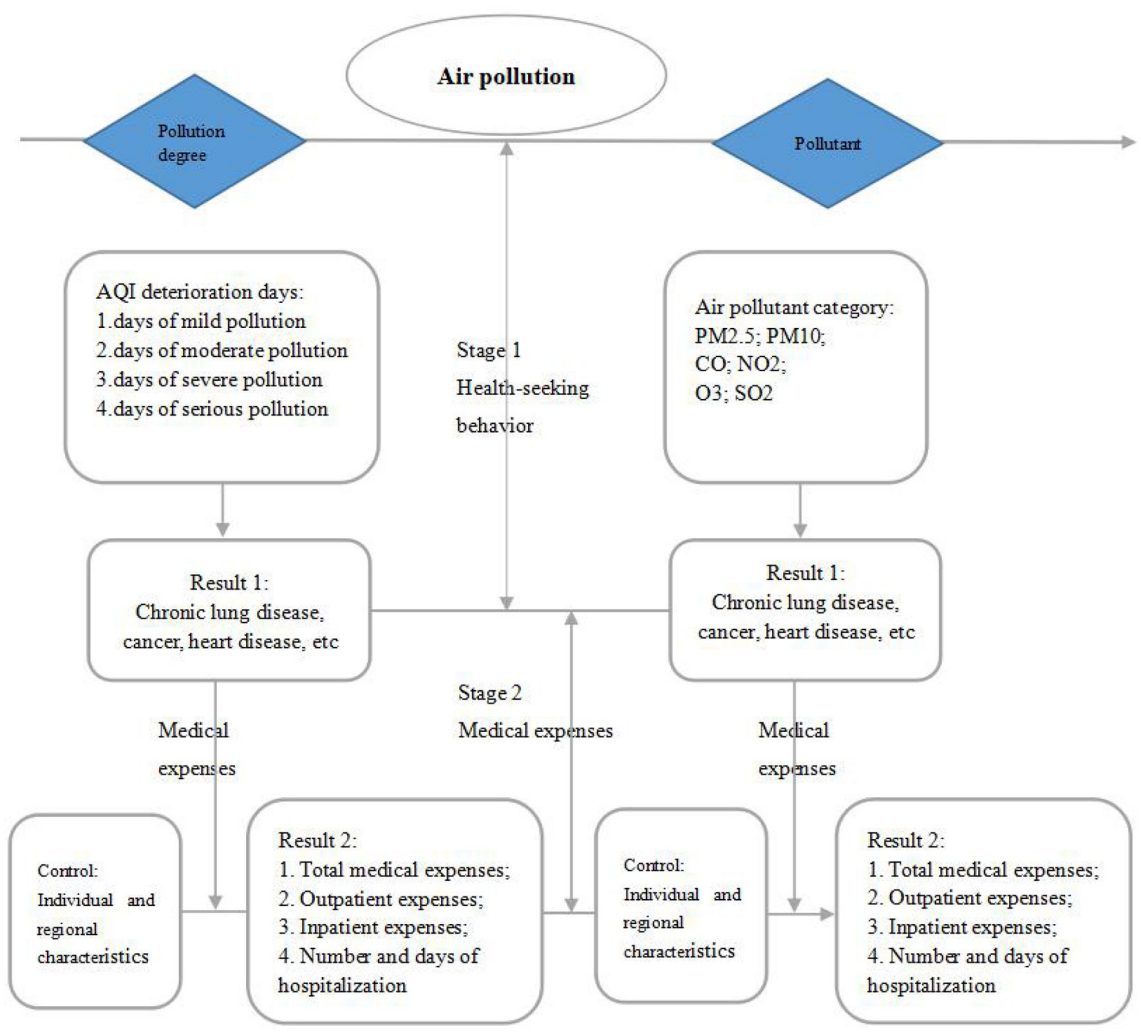
Heckman two-stage model (data source: made by author).

In the second stage of the medical expenses effect model, we used logarithm processing to smoothen the discrete characteristics of the data distribution to promote its normal distribution followed by a linear regression model. The details of the Heckman two-stage model path are shown in Figure 1.

### Endogenous Treatment of Air Pollution

As the endogenous effect of air pollution on residents' medical expenses, for example, may involve reverse causality, the basic data of this study were obtained by matching the CHARLS micro survey data with various regions' annual AQI macro data. Theoretically, there is no causal relationship between reverberations. However, because individual regional health constitutes the overall regional health level, it affects regional socio-economic development to an extent, thus affecting regional air pollution. Second, there were measurement errors and missing variables. It is difficult to avoid recording errors or inaccurate answers in the first-hand data survey because of the public survey data used here. Additionally, as many factors affect health, it is difficult to include all of the possible influencing factors. Therefore, although we had absolute control over the model, there is a possibility of estimation bias.

Based on the benchmark model analysis, we attempted to establish an instrumental variable model for endogenous processing to reduce the estimation bias in the results. Some scholars have used exogenous policy changes to select the instrumental variables. For example, Hanna and Oliva (44) used the closure of polluting factories as an instrumental variable and found that air pollution leads to a decline in student attendance. Some scholars have used inversion weather phenomena to process instrumental variables (24, 28, 45). However, compared with these instrumental variables, regional mineral resource endowment can more directly reflect industrial conditions such as regional industrial emissions and have higher reliability (46, 47). Here, we used the framework provided by Liu and Hu (48) as a reference, selected the regional mineral resource endowment as the instrumental variable of air pollution, and used the two-stage least squares method (2SLS). Theoretically, the selection of instrumental variables should meet the requirements of relevance and exclusivity. First, regional mineral resource endowment is the main measurement index that reflects regional mineral resources and industrial production distribution. Existing studies have shown that gas emissions caused by industrial production are an important source of air pollution. Therefore, regional mineral resource endowments and air pollution were correlated. The proportion of mining employees in the total population is considered a proxy variable for regional mineral resource endowment. Second, regional mineral resource endowment does not directly affect health or medical expenses, but directly affects the health of employees engaged in the mining industry; therefore, it meets the requirements of exclusivity. In the first stage of the instrumental variable model test, F values are >10, indicating no weak instrumental variable problem in the mineral resource endowment test of the selected area, which has strong explanatory significance. Based on this, we establish the following instrumental variable model:


(4) $$\begin{array}{l} x_{ci}=\alpha_{0}+\alpha_{1}Mineral\_endowment_{ci}\\ \text{ ​​​ }+\;\alpha_{2}ind_{ci}+city_{i}+\eta_{ci} \end{array}$$

(5) $$\begin{array}{lll} Medical\text{\_}\exp ense_{ci} & = & \beta_{1}+\beta_{2}x_{ci}+\beta_{3}ind_{ci}+city_{i}+\eta_{ci} \end{array}$$

*Mineral*_*endowment*_*ci*_ in Model (4) is the regional mineral resource endowment. Additionally, the homogeneity of members of the same family in the risks of genetic inheritance, living habits, or external air pollution damage can lead to the autocorrelation of family members in health statistics or medical expenses. Therefore, we selected a family level clustering robust standard error for processing the data.

### Variable Definition and Data

#### Individual Micro Data

The data in this study were selected from the survey data of the CHARLS database in 2018. CHARLS covers most areas of China and has conducted multiple follow-up surveys. Therefore, the heterogeneity and size of this sample can reflect the actual health status of China. Since the CHARLS survey data are collected in a phased manner, the published survey data of 2018 contains survey samples from 2017 and supplementary samples from 2018. Therefore, during data matching, we considered the city as the main body, individual medical expenses as the key, and matched the current regional air pollution situation to obtain the final sample.

The core explanatory variables were residents' health and medical expenses. As mentioned previously, we selected four indicators: chronic lung disease, cancer, heart disease, and self-rated health status. Chronic lung disease was determined based on whether the respondents were diagnosed with relevant lung diseases. Cancer was determined based on whether the respondents were diagnosed with any type of cancer. Heart disease was determined based on whether the subject was diagnosed with heart disease. Chronic lung diseases referred to dummy variables for chronic bronchitis, emphysema, and pulmonary heart disease (excluding tumors or cancer). Cancer is a dummy variable for cancer and other malignant tumors, excluding mild skin cancer. Heart disease refers to the dummy variable for heart disease (myocardial infarction, coronary heart disease, angina pectoris, congestive heart failure, etc.). Self-rated health status was recorded according to the individual's actual evaluation of self-health, with options one to five indicating very healthy, healthy, medium, poor health, and very poor health, respectively. Medical expenses were tested using annual total medical expenses, monthly total outpatient expenses, and annual total inpatient expenses. The total annual medical expenses were obtained according to the sum of the total monthly outpatient medical expenses and total annual inpatient expenses. The total monthly outpatient expenses were calculated according to the total monthly outpatient expenses of the respondents, and the annual inpatient expenses were calculated according to the total annual inpatient expenses of the respondents. The index definitions and statistical values of residents' health and medical expenses are listed in Table 1.

**Table 1:** Variable definition and descriptive statistics.

**Variable**	**Definition**	**Mean**	**Std. Dev**.	**Min**	**Max**
AQI	Air Quality Index, Regional average annual AQI	73.1995	18.0134	40	119
Days of mild pollution	Days of 100 ≤ AQI <150 in a year	57.2525	39.0272	0	152.935
Days of moderate pollution	Days of 150 ≤ AQI <200 in a year	12.4708	13.3424	0	70.81
Days of severe pollution	Days of 200 ≤ AQI <300 in a year	4.6009	6.8010	0	29.93
Days of serious pollution	Days of AQI ≥ 300 in a year	0.6144	1.4565	0	8.03
Income	The total annual income of the respondents, including wage income, etc.	12257.43	49,089.06	0	6,000,000
Outpatient fee	Total outpatient expenses in recent month	1712.306	6,030.361	0	130,000
Inpatient fee	Total inpatient expenses in the latest year	27486.51	61,724.42	0	1400000
Medical expenses	Sum of outpatient and inpatient expenses in the survey year	1517.935	15,445.34	0	1,430,000
Chronic lung disease	Whether the subjects who were diagnosed with chronic lung disease was recorded as 1; otherwise, 0	0.0554	0.2288	0	1
Suffering from cancer	Whether the respondents who were diagnosed with cancer was recorded as 1; otherwise, 0	0.0132	0.1143	0	1
Heart disease	Whether the subjects who were diagnosed with heart disease was recorded as 1; otherwise, 0	0.0796	0.2707	0	1
Self-health	According to the subjective evaluation of self-health, 1–5 indicated that self-rated health was getting worse and worse	2.9463	0.9864	1	5
Age	The age of the subjects	58.7379	10.3188	15	115
Education	According to the educational level of the respondents, 1–11, respectively indicated the levels of education from low to high	3.4767	1.9348	1	11
Gender	Male = 1, female = 0	0.4736	0.4993	0	1
Place of residence	Home = 1, institution = 2, hospital = 3	1.0703	0.4483	1	3
Medical insurance	Enjoy social medical insurance = 1, if not = 0	0.9715	0.1663	0	1
Marital status	Widowed = 1, if not = 0	0.1246	0.3302	0	1
Per capita GDP	Ratio of regional annual GDP to resident population at the end of the year	57,482.27	36,729.53	12625.72	185,942.5
Urban green_coverage	Urban green coverage is the ratio of the vertical projected area of various types of green space in the total urban area.	39.9389	5.0225	19.06	67
Mineral endowment	Proportion of mining employees in the total population	0.0027	0.0068	4.09E-06	0.0654
Household register	Urban = 1, rural = 0	0.4045	0.4908	0	1

#### Air Quality Data

The main indicators reflecting air quality include emissions of pollutants in exhaust gas, detection of the concentration of several air pollutants (e.g., SO_2_, NO_2_, and PM10), annual air pollution index (API), and annual air quality index (AQI). Among them, the main pollutants in the exhaust gas are mainly industrial emissions, and the comprehensive reflection of air quality is limited. AQI is based on the original API, adding fine particulate matter (PM2.5), ozone (O_3_), and carbon monoxide (CO) indicators, which have a comprehensive advantage. Its release frequency is once per hour, which is advantageous in selecting the annual average value of AQI for investigation, with a value range of AQI−0-500. The larger the value, the more serious is the pollution. The AQI data were obtained from the Ministry of Ecology and Environment of China, and the control variable data were obtained from the Urban Statistical Yearbook of China, the Statistical Yearbook of Provinces and Cities, and so on. In addition, we simultaneously investigated the impact of the corresponding indicators of air pollution on medical expenses. The corresponding indicators were selected as follows: days of mild pollution, that is, the days when AQI exceeded 100 (AQI≥100); days of moderate pollution, days when the AQI exceeded 150 (AQI≥150); days of severe pollution, days when the AQI exceeded 200 (AQI≥200); and days of serious pollution, days when the AQI exceeded 300 (AQI≥300).

#### Control Variables

In addition to air quality, the main indicators affecting residents' health and medical expenses also include social development characteristics, such as individual socio-demographic characteristics of respondents and the city's macro environmental characteristics. Therefore, it is necessary to introduce the control variables into the model. We also controlled for individual demographic characteristics such as income, gender, age, marital status, and education, and used regional ID as a proxy index to control for regional macro environment characteristics, such as per capita GDP, urban green coverage, household register, and so on (see Table 1 for the definition and descriptive statistics of variables in this study).

The core variable definitions and descriptive statistics of this study are presented in Table 1.

## Results

### Impact of Air Pollution on Residents' Health

The empirical analysis of this study was conducted using Stata 15 statistical software. Table 2 presents the empirical results of the impact of air pollution on residents' health. Models (1)–(3) were tested using a binary logit model. In model (4), the explained variable is the ordered variable of self-rated health, tested using an ordered model. After controlling for the individual characteristics and the effects of region, the results in Table 2 show that air pollution has a significant positive impact on most health statistical indicators, such as chronic lung disease, heart disease, and self-rated health. Additionally, the results showed that air pollution significantly augmented chronic lung and heart diseases and reduced self-rated health levels. This result is consistent with the conclusions of Schlenker et al. (17) and Zhang et al. (18). It also shows that with the increase in short-term and long-term air pollution, residents' chronic lung disease, heart disease, and self-rated health will be significantly affected.

**Table 2:** Air pollution and health status.

**Variables**	**(1)**	**(2)**	**(3)**	**(4)**
	**Chronic lung disease**	**Suffering from cancer**	**Heart disease**	**Self_health**
AQI	0.0069^***^	0.0040	0.0074^***^	0.0027^***^
	(0.0019)	(0.0037)	(0.0016)	(0.0008)
Age	0.0195^***^	0.0251^***^	0.0300^***^	0.0197^***^
	(0.0037)	(0.0071)	(0.0033)	(0.0015)
Edu	−0.0429^**^	0.0124	0.0361^**^	−0.0509^***^
	(0.0205)	(0.0378)	(0.0173)	(0.0080)
Gender	0.3003^***^	−0.3242^**^	−0.4012^***^	−0.2124^***^
	(0.0724)	(0.1389)	(0.0629)	(0.0287)
Place of residence	0.0092	−0.1831	−0.0117	0.0015
	(0.0761)	(0.1991)	(0.0699)	(0.0294)
Medical insurance	0.2077	0.0317	0.1647	0.2270^***^
	(0.2027)	(0.3891)	(0.1847)	(0.0803)
Marital status	0.2200^**^	−0.3043	−0.1829^**^	−0.0867^*^
	(0.0997)	(0.2076)	(0.0928)	(0.0443)
lnlncome	−0.0065	−0.0148	0.0002	−0.0284^***^
	(0.0091)	(0.0174)	(0.0079)	(0.0035)
lnPer capita GDP	−0.1534^**^	0.0198	−0.2339^***^	−0.2219^***^
	(0.0611)	(0.1150)	(0.0528)	(0.0243)
lnUrban green_coverage	−0.1740	2.1778^***^	−0.3727	−0.1807^*^
	(0.2519)	(0.5381)	(0.2276)	(0.1051)
Household register	−0.0667	0.3133^**^	0.2514^***^	−0.1869^***^
	(0.0747)	(0.1385)	(0.0636)	(0.0296)
Constant	−1.3788	−13.6077^***^	−0.6510	-
	(0.9849)	(2.1563)	(0.8860)	-
Regional effect	YES	YES	YES	YES
Log likelihood	−3651.7584	−1310.1801	−4512.3211	−28517.498
Observations	19095	19095	19095	19095

*Standard errors are in parentheses;*

*** *p < 0.01,*

** *p < 0.05, and*

* *p < 0.1*.

### Impact of Air Pollution on Residents' Medical Expenses

#### Benchmark Inspection

The one-stage ordinary least squares (OLS) regression results, as seen in Panel A in Table 3, show that the average AQI and extreme pollution days have a positive, but not significant, impact on residents' health-seeking behavior. Panel B in Table 3 shows the results of using the two-stage Heckman model to correct for selectivity bias, where after controlling for the influence of relevant variables, air pollution significantly increases residents' medical expenses. The marginal effect of the AQI is 0.0059, indicating that for one unit of air pollution deterioration, the burden of residents' medical expenses increases by 0.59%. Regarding extreme pollution days, when the number of days with AQI exceeding 100, 150, 200, and 300 increased by 1 day, the medical expenses of residents increased by 0.23, 0.67, 1.50, and 6.57%, respectively. The coefficients are greater than those in Panel A.

**Table 3:** Air pollution and medical expenses.

**Variables**	**Medical expenses**				
	**(1)**	**(2)**	**(3)**	**(4)**	**(5)**
**A: one stage OLS model (health seeking behavior)**					
AQI	−0.0144				
	(0.0548)				
Days of mild pollution		0.0001			
		(0.0002)			
Days of moderate pollution			−0.0003		
			(0.0007)		
Days of severe pollution				−0.0003	
				(0.0017)	
Days of serious pollution					−0.0113
					(0.0088)
Individual effect	YES	YES	YES	YES	YES
Control	YES	YES	YES	YES	YES
Observations	19095	19095	19095	19095	19095
**B: Heckman two-stage model (medical expenses)**					
AQI	0.0059^**^				
	(0.0027)				
Days of mild pollution		0.0023^***^			
		(0.0009)			
Days of moderate pollution			0.0067^***^		
			(0.0024)		
Days of severe pollution				0.0150^***^	
				(0.0058)	
Days of serious pollution					0.0657^**^
					(0.0307)
Individual effect	YES	YES	YES	YES	YES
Control	YES	YES	YES	YES	YES
Log likelihood	−10978.73	−10977.09	−10985.37	−10985.76	−10986.72
Observations	2203	2203	2203	2203	2203

*Standard errors are in parentheses;*

*** *p < 0.01 and*

** *p < 0.05*.

#### Endogenous Test and Instrumental Variable Treatment

Next, we used regional mineral resource endowment as the instrumental variable to estimate the impact of air pollution on residents' medical expenses. The first-stage results in Table 4 show that the regional mineral resource endowment of models (1)–(5) significantly impacts air pollution. The F value of the first stage is >10, indicating that there is no weak instrumental variable problem; thus, the instrumental variable is effective.

**Table 4:** Instrumental variable treatment: air pollution and medical expenses.

**Variables**	**Explained variable: medical expenses**				
	**(1)**	**(2)**	**(3)**	**(4)**	**(5)**
	**AQI**	**AQI ≥ 100**	**AQI ≥ 150**	**AQI ≥ 200**	**AQI ≥ 300**
**First stage regression results (AQI and extreme pollution days)**					
Mineral resources	583.496^***^ (107.4966)	805.585^***^ (165.563)	365.5741^***^ (79.8171)	305.6618^***^ (55.67559)	61.7463^***^ (16.5070)
First stage F value	37.13	34.62	24.13	24.21	17.29
Control	YES	YES	YES	YES	YES
**Second stage regression results (Medical expenses)**					
AQI	0.0451^**^				
	(0.0187)				
Days of mild pollution		0.0327^**^			
		(0.0140)			
Days of moderate pollution			0.0721^**^		
			(0.0313)		
Days of severe pollution				0.0862^**^	
				(0.0354)	
Days of serious pollution					0.4266^**^
					(0.1988)
Individual effect	YES	YES	YES	YES	YES
Control	YES	YES	YES	YES	YES
Observations	2203	2203	2203	2203	2203

*Standard errors are in parentheses;*

*** *p < 0.01 and*

** *p < 0.05*.

The results show that the AQI still has a significant positive impact on residents' medical expenses, with a marginal effect of 0.0451. For one unit of air pollution deterioration, residents' medical expenses increased by 4.51%. Regarding extreme pollution days, for each additional day with mild, moderate, severe, and serious pollution, residents' medical expenses increased by 3.27, 7.21, 8.62, and 42.66%, respectively. Compared with the benchmark model, each variable's marginal effect is significantly improved under models (1)–(5) to solve the endogenous effects of air pollution, further showing that air pollution has a realistic effect of increasing medical expenses.

From the research results, we can calculate the medical cost effect of air pollution in China. This study shows that when the days of mild, moderate, severe, and serious pollution increased by 1 day, medical expenses increased by 49.6173, 109.4431, 130.8460, and 647.5511 yuan/year, respectively. In 2018, China's total population aged 15 years and above was 1.167 billion, and the corresponding total cost effect of extreme pollution days such as mild, moderate, severe, and serious pollution were 57.9034, 127.720, 152.697, and 755.6921 billion yuan/year, respectively. The conclusions of this study are consistent with those of Liu and Hu (48) and Guan et al. (49) on the health effects of air quality. If China's total 2018 medical expenses were 5912.191 billion yuan, the total medical expenses per unit increase in AQI would have been 266.6398 billion yuan. When the days of mild, moderate, severe, and serious pollution increased by 1 day, the total medical expenses increased by 193.3286, 426.2690, 509.6309, and 2522.1407 billion yuan, respectively. This is similar to the research results of Deryugina et al. (27), that is, the medical cost effect of air pollution is seriously underestimated.

### Robustness Test

Based on the previous analysis, we investigated the heterogeneity of air pollution on residents' medical expenses based on age and gender differences, urban and rural and regional economic development level differences, and different pollutants to improve the robustness of the results.

Tables 5, 6 are based on population heterogeneity. We divided the sample into two groups according to age-−60 years and above, and 60 years and below—to investigate the differences in medical expenses involving air pollution between older and younger people. Table 5 shows that the effect of air pollution on medical expenses for the older population is significantly greater than that for the younger population; the medical expenses of the former (aged 60 years and above) increased by 1.41, 2.98, 6.04, and 33.92%, respectively, with each day of mild, moderate, severe, and serious pollution, respectively.

**Table 5:** Age heterogeneity: air pollution and medical expenses.

**Variables**	**Explained variable: medical expenses**							
	**Age > = 60 years old group**				**Age < 60 years old group**			
	**(1)**	**(2)**	**(3)**	**(4)**	**(5)**	**(6)**	**(7)**	**(8)**
Days of mild pollution	0.0141^*^				0.0206			
	(0.0077)				(0.0146)			
Days of moderate pollution		0.0298^*^				0.0413		
		(0.0163)				(0.0292)		
Days of severe pollution			0.0604^*^				0.0779	
			(0.0322)				(0.0541)	
Days of serious pollution				0.3392^*^				0.4860
				(0.1967)				(0.3787)
Individual effect	YES	YES	YES	YES	YES	YES	YES	YES
Control	YES	YES	YES	YES	YES	YES	YES	YES
Observations	1176	1176	1176	1176	1027	1027	1027	1027
R^2^	0.1808	0.1841	0.1710	0.1391	0.1696	0.1878	0.1801	0.1213

*Standard errors are in parentheses;*

* *p < 0.1*.

**Table 6:** Gender heterogeneity: air pollution and residents' medical expenses.

**Variables**	**Explained variable: medical expenses**							
	**Male group**				**Female group**			
	**(1)**	**(2)**	**(3)**	**(4)**	**(5)**	**(6)**	**(7)**	**(8)**
Days of mild pollution	0.0128				0.0206^**^			
	(0.0095)				(0.0104)			
Days of moderate pollution		0.0296				0.0393^**^		
		(0.0226)				(0.0190)		
Days of severe pollution			0.0649				0.0718^**^	
			(0.0496)				(0.0335)	
Days of serious pollution				0.5019				0.3596^**^
				(0.4584)				(0.1751)
Individual effect	YES	YES	YES	YES	YES	YES	YES	YES
Control	YES	YES	YES	YES	YES	YES	YES	YES
Observations	921	921	921	921	1282	1282	1282	1282
R^2^	0.1584	0.1709	0.1541	0.1091	0.1807	0.1917	0.1921	0.1594

*Standard errors are in parentheses;*

** *p < 0.05*.

Regarding gender differences, Table 6 shows that the impact of air pollution on female residents' medical expenses is significant. Their medical expenses increased by 2.06, 3.93, 7.18, and 35.96%, respectively, for mild, moderate, severe, and serious pollution, respectively; however, the impact of air pollution on medical expenses for males was not significant, and the consumption effect was also lower. This shows a significant gender difference in the impact of air pollution on medical expenses.

Tables 7, 8 are based on urban-rural heterogeneity. Table 7 shows that air pollution has a significant impact on the medical expenses of urban and rural residents. Compared with rural residents, the marginal effect of air pollution on urbanites' medical expenses was higher. With every additional day of mild, moderate, severe, and serious pollution, their medical expenses increased by 2.19, 4.39, 11.55, and 70.66%, respectively. Under the same conditions, rural residents' medical expenses increased by 2.13, 4.16, 7.26, and 40.95%, respectively. These results show that for regional heterogeneity, the impact of air pollution on urban residents' medical expenses is significantly higher than that of rural residents.

**Table 7:** Urban and rural heterogeneity: air pollution and medical expenses.

**Variables**	**Explained variable: medical expenses**							
	**Urban group**				**Rural group**			
	**(1)**	**(2)**	**(3)**	**(4)**	**(5)**	**(6)**	**(7)**	**(8)**
Days of mild pollution	0.0219				0.0213^*^			
	(0.0138)				(0.0129)			
Days of moderate pollution		0.0439^*^				0.0416^*^		
		(0.0250)				(0.0249)		
Days of severe pollution			0.1155^*^				0.0726^*^	
			(0.0637)				(0.0424)	
Days of serious pollution				0.7066^*^				0.4095
				(0.3931)				(0.2645)
Individual effect	YES	YES	YES	YES	YES	YES	YES	YES
Control	YES	YES	YES	YES	YES	YES	YES	YES
Observations	907	907	907	907	1296	1296	1296	1296
R^2^	0.2402	0.2039	0.1433	0.1491	0.1409	0.1825	0.2100	0.1332

*Standard errors are in parentheses;*

* *p < 0.1*.

**Table 8:** Regional economic heterogeneity: air pollution and medical expenses.

**Variables**	**Explained variable: medical expenses**							
	**Economically developed areas**				**Economically underdeveloped areas**			
	**(1)**	**(2)**	**(3)**	**(4)**	**(5)**	**(6)**	**(7)**	**(8)**
Days of mild pollution	0.0062				0.0395			
	(0.0073)				(0.0246)			
Days of moderate pollution		0.0160				0.0643^*^		
		(0.0188)				(0.0360)		
Days of severe pollution			0.0397				0.1230^*^	
			(0.0469)				(0.0670)	
Days of serious pollution				0.2005				0.6506
				(0.2364)				(0.4121)
Individual effect	YES	YES	YES	YES	YES	YES	YES	YES
Control	YES	YES	YES	YES	YES	YES	YES	YES
Observations	741	741	741	741	1462	1462	1462	1462
R^2^	0.2899	0.3098	0.2685	0.2041	0.2274	0.2523	0.2449	0.1463

*Standard errors are in parentheses;*

* *p < 0.1*.

Table 8 shows the impact of air pollution on the heterogeneity of regional economic development levels. We selected the total gross domestic product (GDP) of a region as the classification standard. The regions below the average GDP level were defined as economically underdeveloped regions while the regions higher than the average GDP level were defined as economically developed regions. The heterogeneity test results of the regional economic development level show that the effect of moderate and severe air pollution on medical expenses in economically underdeveloped regions is significantly higher than that in developed regions. With every additional day of moderate and severe pollution, the medical expenses in economically underdeveloped areas increased by 6.43 and 12.30%, respectively. However, the impact of air pollution on medical expenses in economically developed areas was relatively insignificant, and the effect was lower. Furthermore, with the adjustment and upgrade of China's industrial layouts, most highly polluting enterprises were gradually transferred to the inland or economically underdeveloped regions, thus enhancing air pollution in these regions. Additionally, economically underdeveloped regions show the advantages of resource endowment. However, with socio-economic development and the depletion of mineral resources, these traditionally economically developed regions face environmental damage and severe income challenges, such as underemployment, showing the effect of air pollution on medical expenses.

Finally, we investigated the impact of air pollution on medical expenses based on different pollutants using AQI statistics. Table 9 presents the results of this study. To investigate the impact of different pollutants on medical expenses, the number of inpatients and pollution days were further increased. From models (1)–(5) in Table 9, it can be seen that PM2.5, CO, NO_2_, O_3_, and SO_2_ have significant effects on medical expenses, while PM10 has no significant effects. Among them, CO concentration has a significant positive effect on medical and inpatient expenses. As the CO pollution concentration increased by one unit, the inpatient fee increased by 62.49%. As the NO_2_ pollution concentration increased by one unit, the number of annual inpatient days increased by 2.46%. When O_3_ concentration increased by one unit, the medical expenses, inpatient expenses, outpatient expenses, and annual inpatient times increased by 0.74, 0.66, 0.51, and 0.14%, respectively. Additionally, these results show that for different pollutants, there are significant differences in the impact on medical expenses, among which O_3_ concentration has the most significant impact. The other pollutants had a relatively small impact.

**Table 9:** Heterogeneity of different pollutants: air pollution and medical expenses.

**Variables**	**(1)**	**(2)**	**(3)**	**(4)**	**(5)**
	**Medical expenses**	**Inpatient fee**	**Outpatient fee**	**Time_inp**	**Day_inp**
PM2.5	−0.0130	0.0173^*^	0.0091	−0.0017	0.0014
	(0.0096)	(0.0098)	(0.0094)	(0.0022)	(0.0110)
PM10	0.0094	0.0091	−0.0064	0.0018	0.0017
	(0.0066)	(0.0067)	(0.0064)	(0.0019)	(0.0077)
CO	−0.2218	0.6249^**^	0.3739	−0.0256	0.0058
	(0.2795)	(0.2948)	(0.3527)	(0.0471)	(0.3978)
NO_2_	−0.0050	−0.0006	−0.0078	−0.0005	0.0246^*^
	(0.0076)	(0.0063)	(0.0074)	(0.0018)	(0.0126)
O_3_	0.0074^**^	0.0066^**^	0.0051^*^	0.0014^**^	0.0054
	(0.0030)	(0.0027)	(0.0030)	(0.0007)	(0.0035)
SO_2_	0.0032	0.0131	0.0114	0.0036^**^	0.0263^**^
	(0.0113)	(0.0103)	(0.0131)	(0.0015)	(0.0132)
Individual effect	YES	YES	YES	YES	YES
Control	YES	YES	YES	YES	YES
Observations	2203	2203	2203	2203	2203
R^2^	0.1083	0.1893	0.1718	0.1203	0.1214

*Standard errors are in parentheses;*

** *p < 0.05 and*

* *p < 0.1*.

## Discussion

In this study, we found that air pollution has an important correlation with residents' health and medical expenses. Controlling for individual characteristics, regional characteristics, and other factors, air pollution increased the probability of chronic lung disease, heart disease, and other diseases, and simultaneously reduced the residents' self-rated health level. Consistent with our findings, studies focusing on the concentrations of pollutants such as CO, O_3_, PM2.5, and PM10 in outdoor air pollutants show that an increase in the concentration of these pollutants significantly increases the probability of respiratory diseases and heart diseases (16–18). However, while most of the latest related studies have shown that air pollution significantly affects residents' risk of lung cancer (50, 51), our study found that air pollution does not play a role in residents' risk of cancer, because the statistical indicators of cancer incidence in our study include not only lung cancer but also other cancers, which reduces the significance of the impact of air pollution concentration on residents' diseases. This study's dependent variable contains more disease types than previous research, especially identifying respiratory and cardiovascular diseases directly related to air pollution, which enriches the research conclusions regarding the impact of air pollution on residents' health.

This study also focuses on the impact of air pollution on residents' medical expenses and draws the following conclusions: if other factors remain the same, air pollution has a significant increasing effect on medical expenses, and extreme pollution days have a significant positive effect on medical expenses. This is consistent with the research conclusions regarding the medical consumption effect of existing air pollution (35, 36). However, relevant studies have not investigated the medical consumption effect of air pollution, and the existing research conclusions are not theoretically reliable due to poor consideration of selective deviation in medical expenses. This study is also based on the existing research. First, in terms of research methods, the Heckman two-stage model was selected to reduce the estimation bias caused by sample selection bias. Second, based on the comprehensive air pollution statistical index AQI, we studied the degree of air pollution. That is, taking the occurrence days of different AQI critical values as the core explanatory variable, the advantage here involves investigating not only the static effect of annual air pollution AQI value on medical expenses, but also the dynamic effect of air pollution degree (duration) on residents' medical expenses. The key to the research on air pollution and health cost is the scientificity of data selection and the effectiveness of method identification. Therefore, the difficulties and innovations of this study are reflected in the following points.

First, it is important to select appropriate indicators to measure air pollution and its health and social costs. On the one hand, air pollution comes from primary pollution such as emissions; on the other hand, it mainly comes from secondary pollution after chemical reactions (49). In China, there are two main sources of air pollution. The first is the statistics of industrial pollutant emission data, such as the emission statistics of the industrial “three wastes” in all provinces and cities. However, there were major defects in these statistics. For example, only one pollution is considered, the impact of the pollution degree is not fully reflected, it is reported by enterprises, and the data credibility is low. The second is the dynamic monitoring data published by the Ministry of Ecology and Environment. The data mainly examines the air pollution data of 120 cities in China and are published in real time. This study also utilizes dynamic monitoring data for analysis. At the same time, considering the micro health survey data, this study combines the areas covered in the sample with the dynamic monitoring data of the Ministry of Ecology and Environment, and finally obtains the annual average air pollution and pollution degree of each region, which is also consistent with the selection of air pollution data in many studies, in order to ensure the objectivity and accuracy of air pollution data (52, 53).

In terms of health index measurement, more literature focuses on the health impact of air pollution. The first type of literature explores the impact of air pollution on health status (17, 29, 54). The second type of literature reveals the economic costs caused by air pollution from the perspective of public health (27, 32, 37). However, existing literature still has significant shortcomings. For example, when selecting health statistical indicators, more attention is paid to the investigation of single indicators, such as respiratory diseases. With the progress of medicine and research, the damage of air pollution to physical health also includes the risk of hypertension, diabetes, and liver diseases (55, 56). Therefore, based on this reality, in the selection of health statistical indicators on the basis of respiratory diseases, this study also synchronously selected relevant indicators such as heart disease and self-rated health. Among them, self-rated health, as a comprehensive health statistical index, can truly reflect the overall health status of the respondents in the past year, including physical and mental health. Therefore, the test results are more reliable.

Second, this study not only examines the impact of immediate air pollution on medical consumption, but also examines the impact of long-term pollution on medical consumption. The current micro data statistics on the medical expenses of the respondents mainly investigate the medical consumption of the respondents in the previous month or year (57–59), that is, the real-time impact of air pollution. On this basis, this study simultaneously investigated different pollution intensities and their cumulative days. The advantage of this treatment is that it not only investigated the direct disease cost caused by air pollution, but also included the increase in medical consumption caused by the decline in overall health caused by long-term air pollution. Thus, it can effectively reflect the cumulative health costs associated with long-term air pollution.

Third, we must pay attention to model identification when studying medical expenditures. As a typical example of a sample selection model, the observed medical cost sample refers to the conditional mean given the given medical behavior. In existing studies, many scholars choose the Tobit or Heckman two-stage model for estimation (48, 49, 58). In this study, a two-stage Heckman model was selected for sample selection. The purpose of the first stage is to estimate the individual medical probability or the incidence of medical behavior, and the second stage estimates the impact of air pollution on medical expenses. The advantage of this method is that it can reduce the estimation error caused by the existence of zero medical costs, so as to more accurately evaluate the impact of air pollution. At the same time, in order to reveal which pollution source is the core factor causing the rise of residents' medical expenses, this study simultaneously investigated the impact of different pollutants on residents' medical consumption in order to enrich the research conclusions of existing air pollution and to provide an important basis for environmental policy adjustment.

Fourth, in the process of sample selection, the impact of air pollution on medical consumption is endogenous because of the phenomenon of environmental migration or medical treatment in other places. The existing instrumental variables of air pollution mainly include the days of inversion, air flow coefficient (VC), and regional mineral resource endowment (24, 28, 45, 48, 49). The first two instrumental variables mainly focus on the control of the regional natural environment, while the third focuses on the reflection of regional mineral resources. When the regional natural environment is controlled, the external flow of air pollution under the influence of the natural environment can be effectively reduced, and the air pollution at this time mainly comes from internal release, such as industrial pollution emissions or domestic pollution emissions within the region (48). Therefore, this study uses regional mineral resource endowment as an instrumental variable of air pollution to solve the endogeneity problem. And it is mainly represented by regional mineral practitioners (46, 47), which improves the robustness and reliability of the research conclusions.

However, owing to the complexity of air pollution and health influencing factors, our conclusions have some limitations. First, although AQI covers six air pollutants, it cannot fully reflect the impact of all pollutants. Here, the robustness test involved the medical consumption effect of six pollutants, but it did not sufficiently address the impact of the total amount of air pollution. Second, due to the complexity of health influencing factors, in terms of air pollution's health effect and medical consumption effect, the effect on health is direct and the effect on medical consumption is indirect (60, 61). At the same time, macroscopically, with a country's socioeconomic development, as air pollution gradually increases, the total investment in medical and health care also rises (62, 63). From the perspective of micro individual preferences, due to the diminishing marginal utility of consumption and the increase in income, the greater the life or health value (calculated by the discounted value of future income), the greater the willingness to consume (64). Therefore, it is better to investigate the medical consumption effect of air pollution in combination with the regional socio-economic development level; however, due to data limitations, it is not possible to completely control the regional environment, economic development, and other factors, which is also a limitation of this study.

## Conclusion

Based on the research results, this study draws the following conclusions. (1) In the long run, residents in regions with a higher air pollution index or lower air quality are more likely to suffer from chronic lung disease, heart disease, and other diseases, and their subjective self-health evaluation will show poor results. (2) Air pollution significantly impacts residents' medical treatment probability and medical expenses, and the more serious the air pollution is, the more the residents' medical expenses will increase. (3) Group heterogeneity and regional heterogeneity also play a role in the impact of air pollution on medical expenses. (4) In terms of the impact of air pollutants, O_3_ concentration has the greatest impact on medical expenses, followed by CO and NO_2_ concentrations.

## Data Availability Statement

The original contributions presented in the study are included in the article/supplementary material, further inquiries can be directed to the corresponding author.

## Author Contributions

HL drafted and critically revised the paper for important intellectual content, approved the version to be published, and carried out language retouching and modification. TH and MW made a substantial contribution to the concept and design of the work, data interpretation, and drafted the article. MW undertook the language polishing of the revised draft. All authors contributed to the article and approved the submitted version.

## Funding

This work was funded by National Natural Science Fund of China (71904167), Natural Science Foundation of Zhejiang Province (LQ20G030018), and Zhejiang Philosophy and Social Science Planning Project (20NDQN302YB).

## Conflict of Interest

The authors declare that the research was conducted in the absence of any commercial or financial relationships that could be construed as a potential conflict of interest.

## Publisher's Note

All claims expressed in this article are solely those of the authors and do not necessarily represent those of their affiliated organizations, or those of the publisher, the editors and the reviewers. Any product that may be evaluated in this article, or claim that may be made by its manufacturer, is not guaranteed or endorsed by the publisher.
